# Identification of Pathogenicity-Related Genes in Biofilm-Defective *Acidovorax citrulli* by Transposon Tn5 Mutagenesis

**DOI:** 10.3390/ijms161226076

**Published:** 2015-11-25

**Authors:** Jinyan Luo, Wen Qiu, Lei Chen, Syed Ishtiaq Anjum, Menghao Yu, Changlin Shan, Mehmoona Ilyas, Bin Li, Yanli Wang, Guochang Sun

**Affiliations:** 1 Department of Plant Quarantine, Shanghai Extension and Service Center of Agriculture Technology, Shanghai 201103, China; toyanzi@126.com (J.L.); Chenlei200524@163.com (L.C.); 2 State Key Laboratory of Rice Biology, Institute of Biotechnology, Zhejiang University, Hangzhou 310058, China; wenwen20101010@163.com (W.Q.); ishtiaq@kust.edu.pk (S.I.A.); mhyu@zju.edu.cn (M.Y.); changlin_shan@163.com (C.S.); 3 Department of Zoology, Kohat University of Science and Technology, Kohat 26000, Pakistan; 4 Department of Plant Quarantine, Zhoushan Entry-Exit Inspections and Quarantine Bureau, Hangzhou 310012, China; 5 Department of Biotechnology, University of Sargodha, Sargodha 40100, Pakistan; mehmoona.ilyas@gmail.com; 6 State Key Laboratory Breeding Base for Zhejiang Sustainable Plant Pest and Disease Control, Key Laboratory of Detection for Pesticide Residues, Ministry of Agriculture, Zhejiang Academy of Agricultural Sciences, Hangzhou 310021, China; ylwang88@aliyun.com

**Keywords:** bacterial fruit blotch, Tn5, *Acidovorax citrulli*, pathogenesis, biofilm

## Abstract

Biofilm formation is important for virulence of a large number of plant pathogenic bacteria. Indeed, some virulence genes have been found to be involved in the formation of biofilm in bacterial fruit blotch pathogen *Acidovorax citrulli*. However, some virulent strains of *A. citrulli* were unable to format biofilm, indicating the complexity between biofilm formation and virulence. In this study, virulence-related genes were identified in the biofilm-defective strain A1 of *A. citrulli* by using Tn5 insertion, pathogenicity test, and high-efficiency thermal asymmetric interlaced PCR (hiTAIL-PCR). Results from this study indicated that 22 out of the obtained 301 mutants significantly decreased the virulence of strain A1 compared to the wild-type. Furthermore, sequence analysis indicated that the obtained 22 mutants were due to the insertion of Tn5 into eight genes, including Aave 4244 (cation diffusion facilitator family transporter), Aave 4286 (hypothetical protein), Aave 4189 (alpha/beta hydrolase fold), Aave 1911 (IMP dehydrogenase/GMP reductase domain), Aave 4383 (bacterial export proteins, family 1), Aave 4256 (Hsp70 protein), Aave 0003 (histidine kinase, DNA gyrase B, and HSP90-like ATPase), and Aave 2428 (pyridoxal-phosphate dependent enzyme). Furthermore, the growth of mutant Aave 2428 was unaffected and even increased by the change in incubation temperature, NaCl concentration and the pH of the LB broth, indicating that this gene may be directly involved in the bacterial virulence. Overall, the determination of the eight pathogenicity-related genes in strain A1 will be helpful to elucidate the pathogenesis of biofilm-defective *A. citrulli*.

## 1. Introduction

*Acidovorax citrulli* is the causal agent of bacteria fruit blotch (BFB) in cucurbit plants [1,2]. Since its first outbreak in 1987 [3], BFB has broadened its host range worldwide, together with the lack of effective management methods, making it a potentially serious threat to the cucurbit industry [4,5,6,7,8,9,10]. During the last several decades, the occurrence of this disease has been widely reported, while a number of studies have been carried out for the identification and detection of the bacterial pathogen [11]. For example, the ELISA and real-time PCR methods have been widely applied in the detection of this pathogenic bacterium [12,13]. In addition, our recent research found that the *A. citrulli* strains could be differentiated from related species based on MALDI-TOF MS and Fourier transform infrared (FTIR) spectra [14].

In recent years, more and more studies have focused on the pathogenesis of *A. citrulli*, which was greatly facilitated with the release of the complete genome sequence of the group II strain AAC001-1 of *A. citrulli* in 2007 [15,16]. Indeed, a number of pathogenicity-related genes have been identified in *A. citrulli* [17,18,19]. For example, the genes encoding type III secretion system (T3SS) and type IV secretion system have been found to be required for the virulence, motility, and biofilm formation of *A. citrulli* [20,21]. However, compared to the other studied strains, the virulent strain A1 of *A. citrulli* was defective in biofilm formation, which has been reported to be implicated in the virulence of diverse bacterial pathogens.

Until recently, little information was available about the process of biofilm formation and its regulation due to biofilm formation being a dynamic and complex process involving signal transduction systems, transcriptional regulation, and stress responses [22]. Furthermore, key events that occurred during this developmental process have been described using *Pseudomonas aeruginosa* as a model microorganism. Briefly, flagellar mobility is crucial for approaching the surface, while type IV pili motility is preponderant for surface colonization and microcolonies formation [23]. Obviously, this process is related to bacterial virulence, which makes it necessary to identify the pathogenicity-related genes in the biofilm-defective strain A1 of *A. citrulli*.

The aim of this study was to identify and characterize the genes related to the pathogenicity of biofilm-defective *A. citrulli* based on Tn5 transposon insertion mutations, virulence measurement, and location of transposon by high-efficiency thermal asymmetric interlaced PCR (hiTAIL-PCR) in strain A1 of *A. citrulli*.

## 2. Results and Discussion

### 2.1. Construction of Virulence-Related Mutants

**Figure 1:**
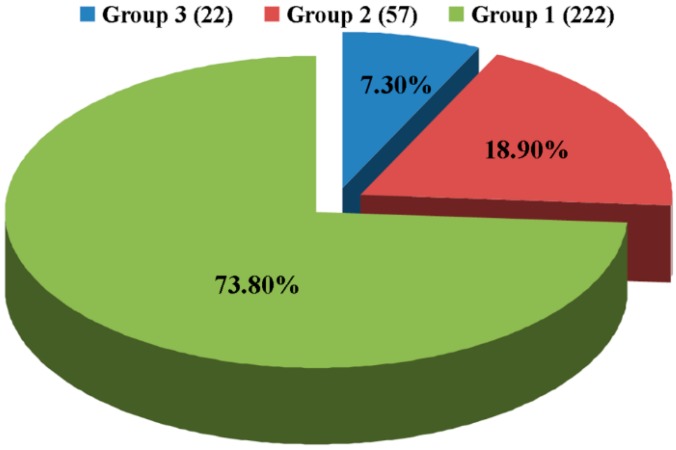
Classification of the Tn5 random insertion mutants based on their virulence to watermelon**.** Group 1 contains 222 mutants with three, four, and five scales of disease severity; Group 2 contains 57 mutants with two scales of disease severity; Group 3 contains 22 mutants with 0 and 1 scale of disease severity. Disease severity index (5, 4, 3, 2, 1 and 0): lesion area was equal to >90%, 73%–90%, 55%–72%, 37%–54%, 19%–36%, and 0%–18% of the wild type, respectively.

Results from this study indicated that random Tn5-inserted mutagenesis generated 301 kanamycin-resistant transconjugants, which were further identified and verified by PCR amplifications using both *A. citrulli*-specific primers AC-F + AC-R and Tn5 primers Kan-F + Kan-R (Table 1). Furthermore, the result of pathogenicity test indicated that there was a difference in the virulence of the 301 mutants, which could be divided into three groups based on the disease severity. In detail, group 1 contains 222 mutants with three, four, and five scales of disease severity, which did not affect, or slightly reduced, the virulence compared to the pathogen control. Group 2 contains 57 mutants with two scales of disease severity, which moderately reduced the virulence compared to the pathogen control. Group 3 contains 22 mutants with 0 and 1 scale of disease severity, which significantly reduced the virulence compared to the pathogen control (Figure 1).

**Figure 2:**
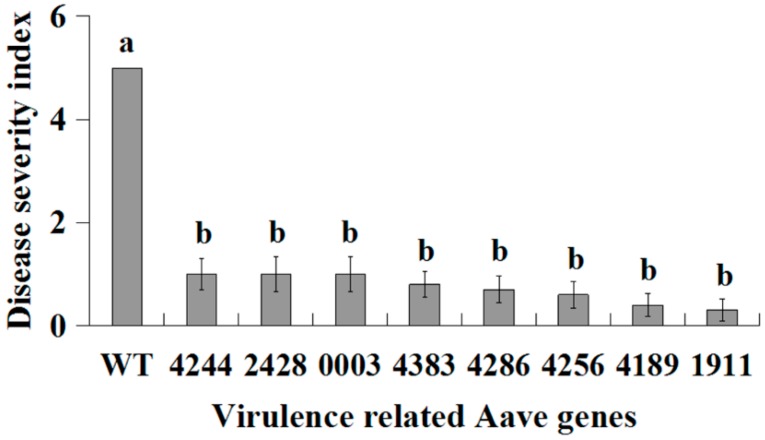
Pathogenicity of the eight mutants to watermelon leaves. Disease severity index was determined as described in Figure 1 and presented as means ± standard errors (*n* = 6). Columns with different letters (a, b) are significantly different according to LSD test (*p* = 0.05).

Further analysis was carried out for the 22 mutants in group 3, which was due to the insertion of the Tn5 into eight genes (as shown in the following section). The transposon insertion frequency varied from 0.052 to 0.068, while each mutant showed insertion stability over at least three generations. Inoculation of watermelon leaves indicated that the virulence was significantly reduced by the mutants compared to the pathogen control, while the reduction percentage of disease severity scale varied from 80.0%–94.0% (Figure 2). Furthermore, there was no significant difference in virulence between the mutants of the eight genes. The result in this study clearly indicated that the mutants of the eight genes were involved in the virulence of this biofilm-defective strain A1 of *A. citrulli*.

**Table 1:** Oligonucleotide primers used in this study for high-efficiency thermal asymmetric interlaced PCR.

Primers Name	Sequence (5’-3’)	*T*_m_ (°C)	GC (%)	Characteristics of Primers
*AC-F*	GACCAGCCACACTGGGAC	55	67	identification of *A. citrulli* strains
*AC-R*	CTGCCGTACTCCAGCGAT	53	61	
*Kan-F*	TTGTCAGCTTCGGTCAGTTG	52	50	identification of the Tn5 mutants
*Kan-R*	GCCTGAGCGAGACGAAATAC	54	55	
*AD-1*	ACGATGGACTCCAGAGCANANNNGGAA	57	52	amplification of unknown sequence flanking transposon Tn5 in pre-amplification step
*SP-1 (get 3’ sequence)*	TTGCGCCTGAGCGAGACGAAATAC	59	54	
*SP-4 (get 5’ sequence)*	ATCAGATCACGCATCTTCCC	52	50	
*SP-2 (get 5’ sequence)*	ACGATGGACTCCAGATTGATGGTCGGAAGAGGC	67	55	amplification of unknown sequence flanking transposon Tn5 in the primary step
*SP-5 (get 3’ sequence)*	ACCTACAACAAAGCTCTCATCAACC	56	44	
*AD-2*	ACGATGGACTCCAGAG	46	56	amplification of unknown sequence flanking transposon Tn5 in the secondary step
*SP-3 (get 3’ sequence)*	TCGCACCTGATTGCCCGACATTAT	57	50	
*SP-6 (get 5’ sequence)*	AGATGTGTATAAGAGACAG	45	37	

### 2.2. Location of Tn5 Inserted Sites

The transposon insertion sites were located by hiTAIL-PCR of the genomic DNAs extracted from the 22 mutants in group 3. Agarose gel electrophoresis analysis of hiTAIL-PCR products indicated that in the pre-amplification reactions, an average of 3.5 products ranging from 100 to 10,000 bp in size was obtained. The result of secondary TAIL-PCR showed that there were only two bands (caused by the usage of TAIL primers amplifying in different directions) representing the flanking sequences around the inserted transposons. Furthermore, the eight different insertion sites were identified from the 22 *A. citrulli* mutants by comparing their sequences with the *A. citrulli* genome in GenBank using BLAST. This result also reveals that different mutants had the same transposon insertion sites. Indeed, sequence comparison indicated that the eight insertion sites belong to Locus tag: Aave 4244 (three mutants), Locus tag: Aave 4286 (four mutants), Locus tag: Aave 4189 (two mutants), Locus tag: Aave 1911 (one mutant), Locus tag: Aave 4383 (five mutants), Locus tag: Aave 4256 (two mutants), Locus tag: Aave 0003 (four mutants), and Locus tag: Aave 2428 (one mutant).

### 2.3. Functional Prediction of Virulence-Related Genes

The eight genes involved in the virulence of biofilm-defective strain A1 were further characterized based on *in silico* function prediction using Pfam (Table 2). Results indicated that no information was found to be used for function prediction of Locus tag Aave 4286. Locus tag Aave 4244 (a member of cation efflux family) was predicted to raise the tolerance to several divalent metal ions; for example, cadmium, zinc, and cobalt [24]. Locus tag: Aave 4189 was annotated as Abhydrolase-1, which is responsible for alpha/beta hydrolase fold and is common to a number of hydrolytic enzymes [25]. Locus tag Aave 1911 possessed an IMP dehydrogenase and GMP reductase domain, which were involved in purine metabolism [26,27,28]. Locus tag: Aave 4383 was predicted to be a member of Flagellar assembly proteins type three secretion system encoding bacterial export proteins such as FliR, MopE, SsaT, YopT, Hrp, HrcT, and SpaR and all of these members export proteins, that do not possess signal peptides, through the membrane [29]. Locus tag Aave 4256 was predicted to belong to 70-kilodalton heat shock family, which were important for protein folding, namely, played essential roles in protein synthesis, transport and degradation, and can protect cells from stress and apoptosis [30,31,32]. Locus tag: Aave 0003 was annotated as histidine kinase-, DNA gyrase B-, and HSP90-like ATPase family, which existed in several ATP-binding proteins [33]. Locus tag: Aave 2428 was predicted as a member from pyridoxal-phosphate-dependent enzyme family consisting of several dehydratases or synthases related with amino acid metabolism [34,35,36].

**Table 2:** Identification of Tn5 transposon insertion sites by hiTAIL-PCR with BLAST and functional prediction by Pfam 28.0 (May, 2015).

Locus_Tag	Gene Name	Pfam No.	Pfam E-Value	Mutant Nos.	Function Prediction
Aave 4244	*Cation_efflux*	PF01545	1 × 10^39^	3	Cation diffusion facilitator family transporter
Aave 4286	Not found	Not found	–	4	Hypothetical protein
Aave 4189	*Abhydrolase_1*	PF00561	4.2 × 10^16^	2	Alpha/beta hydrolase fold
Aave 1911	*IMPDH*	PF00478	2.1 × 10^62^	1	IMPdehydrogenase/GMP reductase domain
Aave 4383	*Bac_export_1*	PF01311	1.1 × 10^66^	5	Bacterial export proteins, family 1
Aave 4256	*HSP70*	PF00012	5.8 × 10^17^	2	Hsp70 protein
Aave 0003	*HATPase_c*	PF02518	2.2 × 10^18^	4	Histidine kinase, DNA gyrase B, and HSP90-like ATPase
Aave 2428	*PALP*	PF00291	1.7 × 10^42^	1	Pyridoxal-phosphate dependent enzyme

**Figure 3:**
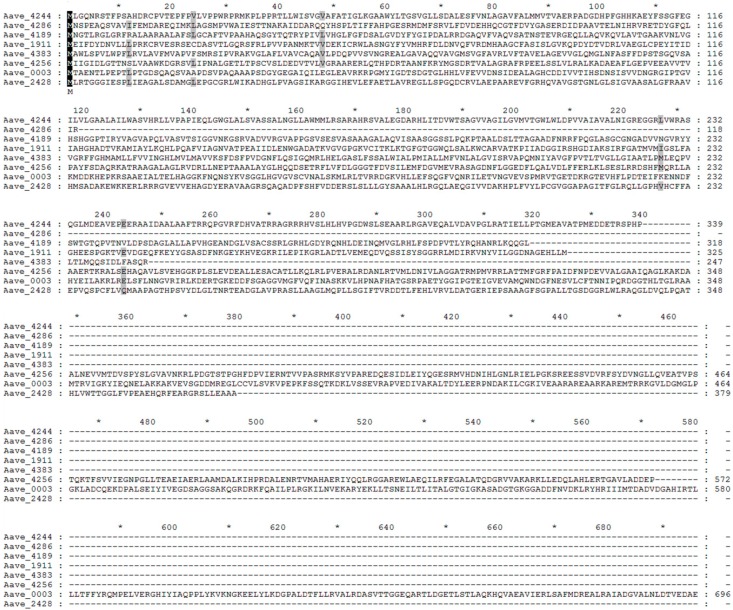
Traditional exploratory visual analysis of multiple sequence alignment. A depiction of the multiple sequence alignment of the predicted proteins of the eight virulence-related genes. *, addition of position by 10 amino acids; black background, the sequences were identical; gray background, the sequences were similar.

Gene function prediction justified the involvement of the eight virulence-related genes in the pathogenesis of biofilm-defective strain A1. For example, Aave 4244 and Aave 4383 were both predicted to be involved in the cells’ membrane secretion processes, which have been reported to be closely connected with bacterial virulence. In agreement with the results of this study, the homologous gene Znts of Aave 4244 [37] has been found to undertake the export of some divalent metal ion out of cells and guarantee cells’ resistance to such ions in *Staphylococcus aureus*. According to [29], Aave 4383’s homology FliQ/R encodes proteins implicated in the export of virulence factors and their mutations in *Caulobacter crescentus* exhibits defects in cell division. After determination of virulence-related genes by transposon mutation, the critical amino acid was able to be clarified by using a point mutation technique based on the predicted protein sequences of the eight genes (Figure 3).

### 2.4. Growth of Virulence-Related Mutants

Results from this study indicated that, compared to the wild-type, Tn5 transposon insertion caused differential change in the growth of *A. citrulli* strain A1 (Table 3). Indeed, the growth of mutant Aave 2428 was unaffected after 12 h of incubation, and even significantly increased by 15.75% after 24 h of incubation compared to the wild-type. Interestingly, the results of virulence testing indicated that mutation of Aave 2428 significantly reduced the pathogenicity to watermelon seedlings compared to the wild type. This result suggests that Aave 2428 may be directly involved in bacterial virulence, not due to the indirect change in bacterial growth.

However, in contrast with the result of Aave 2428, the insertion of Tn5 transposon significantly reduced the growth of mutants Aave 4256, Aave 0003, Aave 4189, Aave 4383, Aave 1911, Aave 4244, and Aave 4286 compared to the wild type regardless of the incubation time (Table 3). Indeed, compared to the wild type, the growth of the seven mutants was reduced by 45.12%–80.49% after 12 h of incubation, while the growth of the seven mutants was reduced by 37.01%–77.17% after 24 h of incubation. This suggests that the reduction in virulence of the seven mutants may be, at least partially, due to the reduction in bacterial growth. In agreement with the result of this study, our recent study found that several genes encoding Type VI secretion system of *Acidovorax avenae* subsp. *avenae* were involved in both bacterial growth and virulence [38]. However, the growth inhibition was excluded from the causes for the reduction in virulence by mutant Aave 2428, revealing the complexity in virulence mechanism of biofilm-defective strain A1.

**Table 3:** Effect of incubation time on the growth of virulence-related mutants of biofilm-defective strain A1 in LB broth.

Bacterial Strains	Incubation Time (h)	
	12	24
Wild type	0.82 ± 0.02 ^a^	1.27 ± 0.02 ^b^
Aave 2428	0.95 ± 0.01 ^a^	1.47 ± 0.01 ^a^
Aave 4256	0.45 ± 0.05 ^b^	0.80 ± 0.02 ^c^
Aave 0003	0.41 ± 0.05 ^b^	0.51 ± 0.04 ^d^
Aave 4189	0.37 ± 0.04 ^b^	0.46 ± 0.04 ^d^
Aave 4383	0.40 ± 0.05 ^b^	0.45 ± 0.04 ^d^
Aave 1911	0.16 ± 0.01 ^c^	0.35 ± 0.01 ^e^
Aave 4244	0.17 ± 0.01 ^c^	0.30 ± 0.01 ^e^
Aave 4286	0.17 ± 0.00 ^c^	0.29 ± 0.01 ^e^

Bacterial growth was measured by optical density at 600 nm (OD_600_). Data from the repeated experiments were pooled and presented as means ± standard error. Treatments with different letters (a–e) are significantly different according to LSD test (*p* = 0.05).

### 2.5. Stress Response of Virulence-Related Mutants

Results from this study indicated that the growth of the wild type and the eight mutants of *A. citrulli* strain A1 were affected by the incubation temperature (Table 4). In general, the wild-type and the eight mutants grew better at 30 °C than that at 15 and 45 °C, while similar growth trends were observed at 15 and 45 °C. However, there was a difference in the growth between the wild-type and the eight mutants at the same temperature of incubation. Indeed, the growth of mutants Aave 4244, Aave 4286, and Aave 1911 at 15 °C was significantly reduced by 81.58%–84.21%, while the growth of the other four mutants at 15 °C was unaffected compared to the wild-type. Furthermore, the growth of mutant Aave 2428 at 30 °C was significantly increased by 15.75%, while the growth of the other seven mutants at 30 °C was significantly reduced by 37.01%–77.17% compared to the wild-type. In addition, the growth of mutant Aave 2428 at 45 °C was significantly increased by 45.71%, the growth of mutants Aave 4244, Aave 4286 and Aave 1911 was significantly reduced by 80.00%–91.43%, while the growth of the other four mutants was unaffected compared to the wild-type. This result indicated that the growth of mutant Aave 2428 was unaffected and even increased regardless of the incubation temperature, which is consistent with the above result of incubation time (Table 3).

Furthermore, the growth of the wild-type and the eight mutants of *A. citrulli* strain A1 in this study were also affected by the concentration of NaCl in LB broth. In general, the wild-type and the eight mutants grew better at 30 °C for 24 h in LB broth with 170 mM NaCl than that with 340 mM NaCl. However, there was a difference in the growth between the wild-type and the eight mutants at the same NaCl concentration (Table 5). Indeed, 170 mM NaCl caused a 15.75% increase in the growth of mutant Aave 2428, and a 37.01%–77.17% reduction in the growth of the other seven mutants compared to the wild-type. Furthermore, the growth of mutant Aave 2428 was unaffected by the 340 mM NaCl, which resulted in a 52.08%–89.58% reduction in the growth of the other seven mutants compared to the wild-type. Interestingly, the study clearly indicated that mutant Aave 2428 has a greater tolerance to salt stress than the other seven mutants. Indeed, the growth of mutant Aave 2428 was unaffected, and even increased, regardless of the NaCl concentration in LB broth.

**Table 4:** Effect of incubation temperature on the growth of virulence-related mutants of biofilm-defective strain A1 in LB broth.

Bacterial Strains	Temperature (°C)		
	15	30	45
WT	0.38 ± 0.04 ^a^	1.27 ± 0.02 ^b^	0.35 ± 0.02 ^b^
Aave 4189	0.38 ± 0.01 ^a^	0.46 ± 0.04 ^d^	0.30 ± 0.03 ^b^
Aave 4383	0.37 ± 0.01 ^a^	0.45 ± 0.04 ^d^	0.25 ± 0.05 ^b^
Aave 0003	0.36 ± 0.02 ^a^	0.51 ± 0.04 ^d^	0.24 ± 0.04 ^b^
Aave 2428	0.34 ± 0.01 ^a^	1.47 ± 0.01 ^a^	0.51 ± 0.03 ^a^
Aave 4256	0.33 ± 0.02 ^a^	0.80 ± 0.02 ^c^	0.20 ± 0.04 ^b^
Aave 4244	0.07 ± 0.00 ^b^	0.30 ± 0.01 ^e^	0.03 ± 0.00 ^c^
Aave 4286	0.07 ± 0.00 ^b^	0.29 ± 0.01 ^e^	0.07 ± 0.05 ^c^
Aave 1911	0.06 ± 0.00 ^b^	0.35 ± 0.01 ^e^	0.07 ± 0.04 ^c^

Bacterial growth was measured by optical density at 600 nm (OD_600_). Data from the repeated experiments were pooled and presented as means ± standard error. Treatments with different letters (a–e) are significantly different according to LSD test (*p* = 0.05).

**Table 5:** Effect of NaCl on the growth of virulence-related mutants of biofilm-defective strain A1 in LB broth.

Bacterial Strains	NaCl	
	170 mM	340 mM
WT	1.27 ± 0.02 ^b^	0.96 ± 0.02 ^a^
Aave 2428	1.47 ± 0.01 ^a^	1.01 ± 0.02 ^a^
Aave 4256	0.80 ± 0.02 ^c^	0.46 ± 0.05 ^b^
Aave 0003	0.51 ± 0.04 ^d^	0.32 ± 0.03 ^c^
Aave 4189	0.46 ± 0.04 ^d^	0.32 ± 0.03 ^c^
Aave 4383	0.45 ± 0.04 ^d^	0.35 ± 0.03 ^bc^
Aave 1911	0.35 ± 0.01 ^e^	0.15 ± 0.01 ^d^
Aave 4244	0.30 ± 0.01 ^e^	0.14 ± 0.02 ^d^
Aave 4286	0.29 ± 0.01 ^e^	0.10 ± 0.01 ^d^

Bacterial growth was measured by optical density at 600 nm (OD_600_). Temperature (30 °C) and incubation time (24 h) were used as independent parameters. Data from the repeated experiments were pooled and presented as means ± standard error. Treatments with different letters (a–e) are significantly different according to LSD test (*p* = 0.05).

Similar to temperature and salt stress, results from this study also indicated that the growth of the wild-type and the eight mutants of *A. citrulli* strain A1 were affected by the pH of LB broth (Table 6). In general, the wild-type and the eight mutants grew better at pH = 7.0 in LB broth than that at pH = 5.0 and pH = 9.0 except that Aave 4244 and Aave 4286 grew better at pH = 5.0 in LB broth than that at both pH = 7.0 and pH = 9.0. However, there was a difference in the growth between the wild-type and the eight mutants at the same pH. Indeed, at the pH of 5.0, the growth of mutant Aave 2428 was significantly increased by 26.56%, the growth of mutants Aave 4244 and Aave 4286 was unaffected, and the growth of the other five mutants was significantly reduced by 62.77%–84.04% compared to the wild type. Furthermore, at the pH of 7.0, the growth of mutant Aave2428 was significantly increased by 15.75%, while the growth of the other seven mutants was significantly reduced by 37.01%–77.17% compared to the wild-type. In addition, at the pH of 9.0, the growth of mutant Aave 2428 was unaffected, while the growth of the other seven mutants was significantly reduced by 47.27%–90.91% compared to the wild-type (Table 6). This result clearly revealed that the growth of mutant Aave 2428 was unaffected and even increased compared to the wild type regardless of the change of the pH of LB broth, which is consistent with its higher tolerance to the change of temperature and salt stress.

**Table 6:** Effect of pH on the growth of virulence-related mutants of biofilm-defective strain A1 in LB medium.

Bacterial Strains	pH		
	5.0	7.0	9.0
WT	0.94 ± 0.19 ^b^	1.27 ± 0.02 ^b^	1.10 ± 0.02 ^a^
Aave 2428	1.28 ± 0.02 ^a^	1.47 ± 0.01 ^a^	1.21 ± 0.07 ^a^
Aave 4256	0.31 ± 0.03 ^c^	0.80 ± 0.02 ^c^	0.58 ± 0.06 ^b^
Aave 0003	0.26 ± 0.06 ^c^	0.51 ± 0.04 ^d^	0.34 ± 0.03 ^cd^
Aave 4189	0.15 ± 0.06 ^c^	0.46 ± 0.04 ^d^	0.32 ± 0.05 ^cd^
Aave 4383	0.19 ± 0.04 ^c^	0.45 ± 0.04 ^d^	0.40 ± 0.05 ^bc^
Aave 1911	0.35 ± 0.04 ^c^	0.35 ± 0.01 ^e^	0.26 ± 0.04 ^de^
Aave 4244	1.00 ± 0.03 ^ab^	0.30 ± 0.01 ^e^	0.10 ± 0.02 ^e^
Aave 4286	0.70 ± 0.02 ^b^	0.29 ± 0.01 ^e^	0.24 ± 0.05 ^de^

Bacterial growth was measured by optical density at 600 nm (OD_600_). Temperature (30 °C) and incubation time (24 h) were used as independent parameters. Data from the repeated experiments were pooled and presented as means ± standard error. Treatments with different letters (a–e) are significantly different according to LSD test (*p* = 0.05).

## 3. Experimental Section

### 3.1. Bacterial Strains and Growth Conditions

Biofilm-defective strain A1 of *A. citrulli* used in this study was provided by Chinese Academy of Agricultural Sciences, China, which was originally isolated from diseased leaf of watermelon. The identity of bacterial strain was confirmed using PCR with specific primer pair AC-F + AC-R for *A. citrulli* [14], while the biofilm formation of this bacterial strain was determined by using the crystal dye method [39]. Strain A1 was routinely grown either on LB agar plate or in LB broth at 30 °C overnight with shaking at 160 rpm until the mid-log phase.

### 3.2. Transposon Insertion of Tn5

After pre-cooled for 30 min, bacterial cells were suspended in 1 mL sterile 10% glycerol to an OD_600_ value of 0.3 to 0.4. Competent cell preparation and electroporation transformation were conducted using a previously published protocol [17,40], with a few modifications. Firstly, 15% glycerol and SOC media were replaced by 10% glycerol and LB media, respectively, while at a lower centrifuge speed at 5000 rpm and lower pulse at 25 μF were used in this study; Secondly, 1 μL of the Tn5 transposon (Epicentre, WI, USA), instead of 10 ng of plasmid DNA or other linear DNA was added to the prepared cells; Thirdly, in order to obtain the highest transformation efficiencies, 2500 volts and 30 °C were utilized as described by Wang *et al.* [40]. PCR amplifications were carried out using genomic DNA isolated from different colonies to detect transposon Tn5 mutagenesis with primer pairs Kan-F and Kan-R, which were listed in Table 1.

### 3.3. Virulence of Mutants to Watermelon

The virulence to watermelon was evaluated based on the needle puncturing method [41], which was carried out by inoculating the leaves of watermelon seedling with the wild-type and the mutants. The mutants were grown on LB medium containing 50 μg/mL kanamycin and then grown at 30 °C with shaking at 200 rpm for 16 h. Pins impregnated with inoculum were used to stab watermelon leaves, while the wild type of strain A1 and LB medium were used as a positive and negative control, respectively. Watermelon seedlings were kept in an incubator for five days in a day/night temperature of 30/25 °C, then the diseased incidence in leaves were calculated. Disease severity index was determined as described by Jiang *et al.* [42] with minor modification based on shoot-area of inoculated leaves relative to the pathogen control: five means lesion area >90% of the pathogen control, while four to zero means that lesion area was within 73%–90%, 55%–72%, 37%–54%, 19%–36%, and 0–18% of the pathogen control, respectively. The experiment was repeated three times, while each treatment consists of six replicates.

### 3.4. Cloning of Tn5-Tagged Regions by hiTAIL-PCR

Genomic DNAs for high-efficiency thermal asymmetric interlaced PCR (hiTAIL-PCR) were extracted from different virulence-related mutants according to the manufacturer’s instructions of TIANamp Bacteria DNA Kit (TIANGEN, Beijing, China). TAIL-PCR including pre-amplification, primary TAIL-PCR and secondary TAIL-PCR was performed as described by [43]. The detailed conditions for hiTAIL-PCR were listed as follow. Pre-amplification: one cycle of 94 °C, 1 min; 98 °C, 1 min; five cycles of 94 °C, 30 s; 65 °C, 1 min; 72 °C, 4 min; one cycle of 94 °C, 30 s; 25 °C, 3 min; 72 °C, 3 min; 15 cycles of 94 °C, 30 s; 61 °C, 1 min; 72 °C, 3 min; 94 °C, 30 s; 61 °C, 1 min; 72 °C, 3 min; 94 °C, 30 s; 58 °C, 1 min; 72 °C, 3 min; and then one cycle of 72 °C, 10 min. Primary TAIL-PCR consists of 13 cycles of 94 °C, 30 s; 65 °C, 1 min; 72 °C, 3 min; 94 °C, 30 s; 65 °C, 1 min; 72 °C, 3 min; 94 °C, 30 s; 50 °C, 1 min; 72 °C, 3 min and one cycle of 72 °C, 10 min; Secondary TAIL-PCR consists of 18 cycles of 94 °C, 30 s; 58 °C, 1 min; 72 °C, 3 min; 94 °C, 30 s; 58 °C, 1 min; 72 °C, 3 min; 94 °C, 30 s; 50 °C, 1 min; 72 °C, 3 min; one cycle of 72 °C, 10 min.

Specific primer sets complementary to the sequences flanking the cloning site of the Tn5 are shown in Table 1. In addition, two AD primers were used in combination with the specific primers for TAIL-PCR [38]. The specific primers were designed to have higher Tms (60–67 °C) than those (*ca.* 50 °C) of AD primers. SP1-6 is specific to Tn5 Transposome (Epicentre, WI, USA). SP1-3 were used to sequence 3′ unknown sequence, while SP4-6 were used for getting anti-sense 3′ unknown nucleic acid. AD1-2 is arbitrary degenerate primers. AD1 was act on pre-amplification, primary TAIL-PCR matching into pairs with SP1 (SP4), SP2 (SP5); AD2 was working on secondary TAIL-PCR in combination with SP3 (SP6). In this PCR reaction system, Tag Premix-Red 2X was purchased from Shanghai biocolor bioscience and Technology Company Limited (Shanghai, China), while the primers were synthesized by Shanghai Sangon Biological Engineering Technology and Service Co., Ltd. (Shanghai, China).

### 3.5. Identification of Virulence-Related Genes

After the secondary TAIL-PCR, the PCR amplified products were analyzed on 0.8% agarose gels, and each single band was recovered and purified from the gels using an AxyPrep DNA Gel Extraction Kit according to the instructions of the manufacturer. The purified productions were sequenced by Shanghai Sangon Biological Engineering Technology and Service Co., Ltd. (Shanghai, China). The obtained sequences of each mutant were compared with the genome of *A. citrulli* strain AAC00-1 (GenBank NC_008752.1) based on Basic Local Alignment Search Tool (BLAST) [44]. Furthermore, the *in silico* functional annotation of virulence-related genes was performed by using Pfam 28.0 (May 2015) [45].

### 3.6. Growth Measurement of the Wild Type and Mutants

A single colony of the wild-type and mutants was picked up and inoculated into 5 mL of LB broth [46] with shaking at 160 rpm at 30 °C overnight. After centrifugation at 6000 rpm for 5 min, the harvested pellets were washed in sterile water twice, and then the finil concentration was adjusted to OD_600_ = 0.30. Bacterial growth was measured by inoculated the mixture of 2 μL of each bacterial suspension with 198 μL of LB broth into individual wells of polystyrene 96-well plates (Commercially available pre sterilized, polystyrene, flat-bottom). After incubation without shaking for 12 h and 24 h at 30 °C that is known to be optimal for bacterial growth, the plates were read by a micro plate reader (Thermo Fisher Scientific Inc., Waltham, MA, USA) at 600 nm. Plates inoculated with the same volume of LB broth served as a negative control. Each treatment had six replicates and this experiment was repeated twice.

### 3.7. Growth of Mutants under Different Conditions

Effect of incubation temperature, pH and salt concentration on the growth of the virulence-related mutants was also determined in commercially available 96-well microtitre plates as described above. Briefly, the effect of pH was examined by inoculating bacteria into LB broth of pH = 5.0, 7.0 and 9.0 at 30 °C for 24 h. The effect of salt concentration was examined by inoculating bacteria into LB broth amended with 170 and 340 mM NaCl, respectively, at 30 °C for 24 h. Furthermore, the effect of incubation temperature on the growth of mutants was determined by incubating bacteria at 15, 30, and 45 °C, respectively, for 24 h. Each treatment had six replicates and this experiment was repeated twice.

### 3.8. Statistical Analyses

The software STATGRAPHICS Plus, version 4.0 (Manugistics Inc., Rockville, MD, USA) was used to perform the statistical analyses. Levels of significance (*p* < 0.05) of main treatments and their interactions were calculated by analysis of variance after testing for normality and variance homogeneity.

## 4. Conclusions

Results from this study indicated that 301 mutants were generated based on random Tn5 insertion mutagenesis, which were further confirmed by PCR amplifications using both *A. citrulli*-specific primers and Tn5 primers. Furthermore, the eight virulence-related genes were identified based on hiTAIL-PCR, BLAST sequence search, and Pfam function prediction of the Tn5 transposon insertion sites from the 22 mutants, which significantly reduced the virulence to watermelon compared to the wild type. In addition, the eight virulence-related mutants differed in the growth and the response to change of temperature, salt and pH stress compared to the wild type of strain A1. Therefore, it could be inferred from this study that the growth condition such as medium composition and temperature should be considered in screening of transposon mutants, while the reduction in virulence of biofilm-defective *A. citrulli* may be due to either the indirect effect by reducing the growth or direct change in virulence. It is very necessary to further validate the phenotypic change by using other approaches such as knockout mutation, however, this study first revealed the potential role of the eight genes in the virulence of biofilm-defective bacteria.
